# Supplementary material for: Perceptions of COVID-19 Maternal Vaccination among Pregnant Women and Healthcare Workers and Factors That Influence Vaccine Acceptance: A Cross-Sectional Study in Barcelona, Spain
Source: Vaccines (Basel). 2022 Nov 15;10(11):1930. doi: 10.3390/vaccines10111930 (PMC9692476; doi:10.3390/vaccines10111930)
Supplement: Supplementary file 1 [file vaccines-10-01930-s001.zip › vaccines-1998563-supplementary.pdf]

Supplementary Material

Survey S1. Survey of Pregnant Women: Knowledge and Perceptions of Maternal Vaccines (English Version)

Hello, mum! From the Barcelona Institute of Global Health, in collaboration with the Hospital Clínic de Barcelona and the ASSIR network, we would like to invite you to participate in the following research study, "Barriers and facilitators for the acceptability of vaccines during pregnancy", to find out more about the perceptions and knowledge about vaccines administered during pregnancy. This is an anonymous survey, so no personal data that can identify you will be collected. Thank you very much for your help!

Data policy: I confirm that I have read the information and understand that my answers will be used for research, maintaining anonymity and without using personal data.

1. What is your age? (_ _)

2. What is your postcode? (_ _ _ _ _)

3. What is your country of origin? (______________)

4. If the answer is different from "Spain", in what year did you arrive in Spain? (_ _ _ _)

5. What is the highest level of education you have completed?

    a) Primary education

    b) Secondary education

    c) Vocational training

    d) Higher education (university degree, bachelor's degree, degree)

    e) Postgraduate studies (master's, doctorate)

6. What is your current occupation?

    a) Employed

    b) Self-employed

    c) Unemployed, looking for work

    d) Unemployed but not currently looking for work

    e) Student

    f) Housewife

    g) Other:____________

7. What week of pregnancy are you in?

    a) Less than 12 weeks

    b) 12-23 weeks

    c) 24-37 weeks

    d) More than 37 weeks

8. Are you currently on maternity leave? Yes or no

9. What is your marital status?

a) Single

b) Married

c) In a relationship

d) Divorced or separated

e) Widowed

10. Have you been pregnant before?

a) No, this is my first pregnancy

b) Yes, once

c) Yes, twice

d) Yes, three or more times

11. How many children do you have? (_)

12. Where do you carry out the control of your current pregnancy? (Please select all that apply)

a) Maternity Hospital Clinic of Barcelona

b) Maternity Hospital Sant Joan de Déu

c) CAP Manso

d) CAP Numancia

e) Other CAP

f) Private clinic

g) Other centre (indicate which one): ______________

13. Do you know of any recommended vaccines for pregnant women? Yes or no

14. Do you know which vaccines are currently recommended during pregnancy? (Please select all that apply)

a) Influenza

b) Whooping cough (Tdap: tetanus, diphtheria and pertussis)

c) COVID-19

d) RSV (respiratory syncytial virus - bronchiolitis)

e) I don't know

15. Have you been vaccinated in any of your previous pregnancies? Yes or no

16. If you have been vaccinated in previous pregnancies, what vaccines did you receive?

a) Pertussis (Tdap: tetanus, diphtheria and pertussis)

b) Influenza

c) None of the above

d) I don't know

e) Others:____________

17. Regarding the vaccines recommended during pregnancy, which do you consider to be reliable sources of information? (Please select all that apply)

a) Midwife

b) Gynaecologist

c) Family doctor

d) Paediatrician

e) Nurse

f) Your partner

g) Relatives

h) Friends

i) Internet

j) News/media

k) Social networks

i) Other mothers

m) Religion

n) Others:____________

18. Do you think that the information received through the Internet about vaccinations during pregnancy is… (Please select all that apply)

a) Trustworthy

b) Incomplete

c) Objective

d) Discouraging

e) Difficult to understand

f) I have never seen information about vaccines on the Internet

g) I don't know

h) Others:____________

19. Did you know that babies during the first months of life can be protected against infectious diseases that are more frequent in that period through vaccinations received by their mothers during pregnancy? Yes or no

20. Please complete the sentence with all the endings that you consider true: "Some vaccines during pregnancy are..." (Please select all the answers that apply)

a) Safe and important for me

b) Safe and important for my baby

c) Effective

d) Good for the general population

e) Compatible with my ethical values/religion

f) I don't know

g) Others:___________

21. Has any of your children been hospitalized for a respiratory infection during the first year of life? Yes or no

22. Has any of your children been hospitalized for sepsis during the first year of life? Yes or no

23. During your current pregnancy, would you be willing to receive the vaccine against... (Please select all that apply)

a) Pertussis (Tdap: tetanus, diphtheria and pertussis)

b) Influenza

c) COVID-19

d) Any vaccines recommended by a healthcare professional

e) None

f) I don't know

24. During your current pregnancy, have you received a flu shot?

a) Yes [if you select this, the survey continues at question 25]

b) No [if you select this, the survey continues at question 26]

c) I don't know [if you select this, the survey goes to question 26]

25. If you have had a flu shot during this pregnancy, what motivated you to get vaccinated? (Please select all that apply)

a) The recommendation of a health professionals

b) The recommendation of my family and friends

c) I think it is good to protect my baby against the flu

d) I think it is good to protect me against the flu

e) Because I know other women who were vaccinated during pregnancy

f) Because it is free

g) Others:__________

26. If you have not been vaccinated against the flu during this pregnancy, what was the reason? (Please select all answers that apply)

a) I haven't been offered a vaccination (it’s too early in pregnancy, it's not flu season...)

b) I have not been told about vaccinations during pregnancy

c) I was afraid that the vaccine would harm me

d) I was afraid that the vaccine might harm my baby

e) Due to time issues (making an extra visit to the hospital, waiting time, distance...)

f) I am against vaccinations during pregnancy

g) I am against vaccinations in general

h) I don't think the flu is dangerous for the baby

i) I don't think the flu is dangerous for me

j) I do not believe that the vaccine is effective

k) Religious/cultural/lifestyle beliefs (please explain under “Other”)

l) Others: ______________

27. During your current pregnancy, have you received the pertussis vaccine (Tdap: tetanus, diphtheria and pertussis)?

a) Yes [if you select this, the survey continues at question 28]

b) No [if you select this, the survey continues at question 29]

c) I don't know [if you select this, the survey goes to question 29]

28. If you have been vaccinated against pertussis (Tdap: tetanus, diphtheria and pertussis) during this pregnancy, what motivated you to get vaccinated? (Please select all that apply)

a) The recommendation of a health professionals

b) The recommendation of my family and friends

c) I think it is good to protect my baby against pertussis (Tdap: tetanus, diphtheria and pertussis)

d) I think it is good to protect me against pertussis (Tdap: tetanus, diphtheria and pertussis)

e) Because I know other women who were vaccinated during pregnancy

f) Because it is free

g) Others:__________

29. If you have not been vaccinated against pertussis (Tdap: tetanus, diphtheria and pertussis) in this pregnancy, what was the reason? (Please select all answers that apply)

a) I have not been offered the vaccine

b) I have not been told about vaccinations during pregnancy

c) I was afraid that the vaccine would harm me

d) I was afraid that the vaccine might harm my baby

e) Due to time issues (making an extra visit to the hospital, waiting time, distance...)

f) I am against vaccination during pregnancy

g) I am against vaccination in general

h) I do not think that whooping cough (Tdap: tetanus, diphtheria and pertussis) is dangerous for the baby

i) I do not think pertussis (Tdap: tetanus, diphtheria and pertussis) is dangerous for me

j) I do not believe that the vaccine is effective

k) Religious/cultural/lifestyle beliefs (please explain under "Other")

l) Others: ______________

30. During your current pregnancy, have you received the COVID-19 vaccine?

a) Yes [if you select this, the survey continues at question 31]

b) No [if you select this, the survey continues at question 32]

c) I don't know [if you select this, the survey continues at question 32]

31. If you have been vaccinated against COVID-19 during this pregnancy, what motivated you to do so? (Please select all that apply)

a) The recommendation of a health professionals

b) The recommendation of my family and friends

c) I think it is good to protect my baby against COVID-19

d) I think it is good to protect me against COVID-19

e) Because I know other women who were vaccinated during pregnancy

f) Because it is free

g) Others:__________

32. If you have not been vaccinated against COVID-19 during this pregnancy, what was the reason? (Please select all answers that apply)

a) I have not been offered the vaccine

b) I have not been told about vaccinations during pregnancy

c) I was afraid that the vaccine would harm me

d) I was afraid that the vaccine might harm my baby

e) Due to time issues (making an extra visit to the hospital, waiting time, distance…)

f) I am against vaccination during pregnancy

g) I am against vaccination in general

h) I don't think COVID-19 is dangerous for the baby

i) I don't think COVID-19 is dangerous for me

j) I do not believe that the vaccine is effective

k) Religious/cultural/lifestyle beliefs (please explain under “Other”)

l) Others: ______________

33. To decide whether or not to get vaccinated during pregnancy, who would you talk to? (Please select all that apply)

a) I would make the decision alone

b) With my partner

c) With my relatives

d) With my midwife

e) With my gynaecologist

f) With my nurse

g) With my family doctor

h) With my other child's paediatrician

i) I would consult information on social networks

i) I don't know

i) Others

34. What information would help you decide to receive the recommended vaccines during pregnancy? (Please select all that apply)

a) Knowing about the safety of vaccines for the baby and me

b) Knowing that vaccines protect the baby

c) Knowing what disease the vaccine protects against

d) Type of vaccine (RNA, inactivated, toxoid vaccine…)

e) I don't know

f) Others:_____________

35. What other reason(s) can help you decide whether to receive the recommended vaccines during pregnancy? (Please select all that apply)

a) Personal determination to protect my child

b) Recommendation by health professionals during pregnancy

c) Knowing that other mothers also get vaccinated

d) My relatives supporting and encouraging me to get vaccinated

e) Being able to get vaccines during my prenatal care visits

36. How much do you agree or disagree with the following statements?

Strongly agree = 5; Somewhat agree = 4; I have some doubts = 3; Somewhat disagree = 2; Strongly disagree = 1

a) I trust the information received by health professionals (gynaecologists, midwives, nurses, family doctors, paediatricians...) regarding maternal vaccines. (_)

b) I am confident that the benefits of vaccines outweigh their risks. (_)

c) If I were offered to participate in a clinical trial of a new vaccine to be administered during pregnancy, I would agree to participate. (_)

37. If you agreed to participate in a clinical trial of a maternal vaccine, would you prefer a vaccine that was:

a) Injected

b) Oral (pill or drops)

c) Nasal spray

d) I don't care about the form of administration

e) None

38. Do you think that vaccinations during pregnancy should be mandatory? Yes or no

39. Indicate the reasons why you would consider that maternal vaccines should be mandatory (Please select all answers that apply)

a) Because they are important in protecting pregnant women, babies, and the entire population from major diseases

b) Because many people are not vaccinated or do not want to be vaccinated

c) Because it is important to prevent the spread of disease throughout the world

d) Because it is important to prevent the reappearance of diseases/infections that are no longer prevalent in our environment

e) Because it is necessary for more women to receive vaccinations during pregnancy, making it mandatory is the only way to increase it.

f) Others: ____________

40. Indicate the reasons why you would consider that maternal vaccines should not be mandatory (Please select all answers that apply)

a) Because the decision to be vaccinated is a personal and voluntary choice

b) Because women must decide based on the risks and frequency of diseases

c) Because I am against the economic benefits that pharmaceutical companies could obtain

d) Others: ____________

41. Do you think this survey will make you reconsider your decision to get vaccinated during your current pregnancy?

a) Yes, before, I was not sure about getting vaccinated, and now I have more information to decide to get vaccinated

b) Yes, before, I wanted to get vaccinated, but now I have doubts.

c) No, my decision has not changed. Before the survey, I already wanted to get vaccinated, and I still want to.

d) No, my decision has not changed. Before the survey, I did not want to get vaccinated, and now I do not want to do it.

e) I don't know

42. If you wish, you can write any comments regarding vaccinations during pregnancy:

____

Survey S2. Survey aimed at health professionals: Knowledge and perceptions about maternal vaccines (Translated from Spanish)

Welcome! From the Barcelona Institute of Global Health, in collaboration with the Hospital Clínic de Barcelona and the Hospital Sant Joan de Dèu, we invite you to participate in the following research study, "Barriers and facilitators for the acceptability of vaccines during pregnancy", to know more about perception and knowledge about vaccines administered during pregnancy. This is an anonymous survey, so no personal data that can identify you will be collected.

Thank you very much for your help.

Data policy: I confirm that I have read the information and understand that my answers will be used for research, maintaining anonymity and without using personal data.

1. Age (_ _)

2. Gender

  a) Female

  b) Male

  c) I prefer not to say

  d) Other: ____________________

3. What is your country of origin? (____________________)

4. What medical speciality do you practice/

  a) Midwife

  b) Nurse

  c) Family medicine

  d) Obstetrics/Gynaecology

  e) Paediatrics

  f) Neonatology

  g) Other: ______________

5. Years of clinical practice:

  a) 1-5

  b) 6-10

  c) 11-15

  d) More than 15

6. What vaccines are you aware of that are currently recommended during pregnancy? (Please select all that apply)

a) Influenza

b) Tetanus

c) Tdap (tetanus, diphtheria and pertussis)

c) COVID-19

e) RSV (Respiratory Syncytial Virus)

f) GBS (Group B Streptococcus)

g) I'm not sure

h) Others:___________

7. Have you received specific training/information, as a professional, about which vaccines are recommended during pregnancy? Yes or no

8. Through what channels have you received training/information about maternal vaccines as a health professional? (Please select all that apply)

a) Courses/specific training on maternal vaccines

b) During my bachelor's degree training

c) During my postgraduate training (speciality, master's, doctorate)

d) Through scientific meetings/congresses, professional societies, or continuing education programmes

e) I have not received specific training about maternal vaccines

f) Others:___________

9. In your daily professional practice, do you usually provide information to pregnant women about maternal vaccines?

a) Yes, almost always

b) Yes, sometimes

c) No

10. Do you recommend pregnant women receive these vaccines in your daily professional practice?

a) Yes, always (the questionnaire continues with question 11)

b) Yes, sometimes (the questionnaire continues with question 11)

c) It depends on the vaccine (the questionnaire continues with question 11)

d) No (the questionnaire continues with question 12)

11. At what time during antenatal care do you first inform pregnant women about influenza vaccination? (Please select all that apply)

a) In the first trimester

b) In the second trimester

c) In the third trimester

d) Depending on the seasonality of the flu

e) I do not inform them about maternal vaccines

f) Other: ____________

12. At what time during prenatal care do you inform pregnant women for the first time about vaccination against whooping cough (Tdap: tetanus, diphtheria and pertussis)? (Please select all that apply)

a) In the first trimester

b) In the second trimester

c) In the third trimester

e) I do not inform them about maternal vaccines

f) Other: ____________

13. How do you provide information to pregnant women about maternal vaccinations? (Please select all that apply)

a) Offering information during antenatal care visits

b) Through brochures/information sheets with specific information on vaccines

c) Through videos/specific online material on vaccines

f) Others:______________

g) I do not inform them about maternal vaccines

14. Do pregnant women refuse to be vaccinated during pregnancy?

a) Always

b) Almost always

c) Sometimes

d) Hardly ever

e) Never

f) Not applicable

15. In the case of women who refuse to receive vaccines during pregnancy, what do you think are the reasons why these women make that decision? (Please select all that apply)

a) Fear of possible adverse effects of the vaccine on the health of their baby

b) Fear of possible adverse effects of the vaccine on their own health

c) They consider that vaccines are neither necessary nor useful in the prevention of diseases

d) They consider it unlikely that they will contract a vaccine-preventable disease

e) Desire to maintain a natural pregnancy

f) General concerns about vaccines

g) Other:_____________

16. If you do not usually recommend maternal vaccines in your professional practice, could you indicate the reasons? (Please select all that apply)

a) Not applicable

b) It is not a part of my medical speciality

c) I am concerned about the safety of the vaccine and possible adverse events

d) The recommended maternal vaccines are for diseases that are no longer common or important

e) The recommended maternal vaccines are not effective enough

f) The recommended maternal vaccines are not as important as other medical controls during pregnancy

g) The workload makes it difficult to recommend and manage vaccinations

h) I only recommend vaccines if the pregnant woman specifically asks about it

17. As a health professional, do you get vaccinated annually to prevent influenza virus infection?

a) Yes, annually

b) Not every year, but I have been vaccinated on some occasions

c) Not every year, but I got vaccinated in 2020

c) No, I have never been vaccinated against the flu

d) Others:_______________

18. If you are a woman and have been pregnant, did you receive any of the following recommended vaccinations during pregnancy? (Please select all that apply)

a) Not applicable

b) Pertussis (Tdap: tetanus, diphtheria and pertussis)

c) Influenza

c) COVID-19

e) No. Please explain the reasons in the free text box under "Others"

f) Others: _________________

19. If your partner was pregnant, would you recommend that she receive the currently recommended vaccines during pregnancy? (Please select all that apply)

a) Not applicable

b) Pertussis (Tdap: tetanus, diphtheria and pertussis)

c) Influenza

c) COVID-19

e) No. Please explain the reasons in the free text box under "Others"

f) Others: _____________

20. How much do you agree or disagree with the following statements?

Strongly agree = 5; Somewhat agree = 4; I have some doubts = 3; Somewhat disagree = 2; Strongly disagree = 1

a) I have all the necessary information on maternal influenza vaccines to recommend these vaccines to pregnant women. (_)

b) I have all the necessary information on maternal pertussis vaccines to recommend these vaccines to pregnant women. (_)

c) I have all the necessary information on maternal vaccines against COVID-19 to recommend these vaccines to pregnant women. (_)

d) Maternal influenza vaccines are safe. (_)

e) Maternal pertussis vaccines are safe. (_)

f) Maternal vaccines against COVID-19 are safe. (_)

g) Maternal influenza vaccines are effective. (_)

h) Maternal pertussis vaccines are effective. (_)

i) Maternal vaccines against COVID-19 are effective. (_)

j) Vaccination against influenza is especially important during pregnancy, and my recommendation to pregnant women is clear in this regard. (_)

k) Vaccination against pertussis is especially important during pregnancy, and my recommendation to pregnant women is clear in this regard. (_)

l) Vaccination against COVID-19 is especially important during pregnancy, and my recommendation to pregnant women is clear in this regard. (_)

m) My recommendation on maternal vaccines may influence pregnant women's decisions. (_)

21. If you wish, you can write any additional comments you want regarding your perception, knowledge or opinions about maternal vaccines:

____
